# Dual regulation by ethanol of the inhibitory effects of ketamine on spinal NMDA-induced pressor responses in rats

**DOI:** 10.1186/1423-0127-19-11

**Published:** 2012-02-02

**Authors:** Nien-Tzu Keng, Hsun-Hsun Lin, Huei-Ru Lin, Wei-Kung Hsieh, Chih-Chia Lai

**Affiliations:** 1 Institute of Medical Sciences, Tzu Chi University, Hualien, Taiwan; 2 Department of Physiology, Tzu Chi University, Hualien, Taiwan; 3 Department of Laboratory Medicine and Biotechnology, Tzu Chi University, Hualien, Taiwan; 4 Department of Pharmacology, Tzu Chi University, Hualien, Taiwan; 5 Department of Radiological Technology, Tzu Chi College of Technology, Hualien, Taiwan; 6 General Education Center, National Taitung Junior College, Taitung, Taiwan

**Keywords:** alcohol, ketamine, NMDA receptor, PKA, phosphorylation, sympathetic neuron

## Abstract

**Background:**

Acute exposure of ethanol (alcohol) inhibits NMDA receptor function. Our previous study showed that acute ethanol inhibited the pressor responses induced by NMDA applied intrathecally; however, prolonged ethanol exposure may increase the levels of phosphorylated NMDA receptor subunits leading to changes in ethanol inhibitory potency on NMDA-induced responses. The present study was carried out to examine whether acute ethanol exposure influences the effects of ketamine, a noncompetitive NMDA receptor antagonist, on spinal NMDA-induced pressor responses.

**Methods:**

The blood pressure responses induced by intrathecal injection of NMDA were recorded in urethane-anesthetized rats weighing 250-275 g. The levels of several phosphorylated residues on NMDA receptor GluN1 subunits were determined by western blot analysis.

**Results:**

Intravenous injection of ethanol or ketamine inhibited spinal NMDA-induced pressor responses in a dose-dependent and reversible manner. Ketamine inhibition of NMDA-induced responses was synergistically potentiated by ethanol when ethanol was applied just before ketamine. However, ketamine inhibition was significantly reduced when applied at 10 min after ethanol administration. Western blot analysis showed that intravenous ethanol increased the levels of phosphoserine 897 on GluN1 subunits (pGluN1-serine 897), selectively phosphorylated by protein kinase A (PKA), in the lateral horn regions of spinal cord at 10 min after administration. Intrathecal administration of cAMPS-Sp, a PKA activator, at doses elevating the levels of pGluN1-serine 897, significantly blocked ketamine inhibition of spinal NMDA-induced responses.

**Conclusions:**

The results suggest that ethanol may differentially regulate ketamine inhibition of spinal NMDA receptor function depending on ethanol exposure time and the resulting changes in the levels of pGluN1-serine 897.

## Background

Ethanol has several effects on the central nervous system, such as intoxication, tolerance, and withdrawal. Although these mechanisms are still not well understood, many evidences suggest an important role of the glutamate neurotransmitter system in ethanol effects [1-3]. It has been repeatedly reported that ethanol antagonizes central effect of glutamate by acting at NMDA (N-methyl-D-asparate) receptors, a subtype of ionotropic glutamate receptors, at pharmacologically relevant concentrations [4,5]. The ability of ethanol to inhibit NMDA-activated current is linearly related to its potency for causing intoxication [6]. NMDA receptors are composed of 7 subunits including a GluN1 subunit, a family of GluN2 subunits (A, B, C, D), and two GluN3 subunits (A and B) [7]. NMDA receptor function is regulated by several kinases and phosphatases. There are several serine residues on GluN1 subunit. The serine residues 896 and 897 on GluN1 subunit are specifically phosphorylated by protein kinase C (PKC) and cAMP-dependent protein kinases (PKA), respectively [8].

Sympathetic preganglionic neurons (SPNs), located in thoracolumbar spinal cord, are the final site where sympathetic activity is integrated within the central nervous system [9]. SPNs provide projections to sympathetic ganglia and adrenal medulla, whose activation elicits an increase in peripheral sympathetic activity and the underlying cardiovascular responses. Our previous studies showed intrathecal injection of NMDA into the T7-T9 segments of spinal cord may cause an increase in blood pressure resulting from the activation of NMDA receptors in SPNs; intravenous injection of ethanol selectively inhibited the NMDA-induced pressor responses [10]. We further demonstrated that prolonged application of ethanol may increase the phosphorylated levels of NMDA receptors by activating signaling pathways and subsequently regulate (counteract) ethanol inhibition of the NMDA receptor function [11], which may contribute to the development of acute ethanol tolerance. We suggest that depending on exposure time and the resulting alteration of the phosphorylated levels of NMDA receptors, acute ethanol may have differential influences on NMDA receptor function. Whether ethanol intake differentially modulates the inhibitory effects of NMDA receptor antagonists on NMDA receptor function remains unclear. The present study was undertaken to examine the hypothesis that acute ethanol exposure may affect the inhibitory effects of ketamine, a non-competitive NMDA receptor channel blocker, on the responses of NMDA receptors in spinal sympathetic neurons using an *in vivo *model established previously; the magnitude of increases in blood pressure induced by intrathecal injection of NMDA was used as an index for responses of NMDA receptors *in vivo*.

## Methods

### Animals

Sprague-Dawley (SD) rats purchased from BioLASCO Co., LTD. (Taipei, Taiwan) were used to establish a breeding colony at the Laboratory Animal Center, Tzu Chi University, Taiwan. Adult male rats weighing 250-270 g selected from the colony were used in the present study. All procedures were carried out in accordance with the guidelines of the Institutional Animal Care and Use Committee of Tzu Chi University. To avoid unnecessary sacrifice and suffering, the number of animal used was minimized, and anesthetics were used throughout the experiment.

### Determination of blood ketamine and ethanol levels

To avoid perturbing the blood pressure recording, blood ketamine and ethanol concentrations were measured in another group of male rats under the same conditions as the experimental ones. The rats were anaesthetized with urethane. The right femoral vein was cannulated for intravenous injection of ketamine (2 mg/kg or 4 mg/kg) or ethanol (0.025 g or 0.16 g). Ketamine (1 ml/kg) or ethanol (1 ml) at known concentrations was injected into the femoral vein in 100 seconds. Blood sample of 0.2 ml was withdrawn from the right femoral artery at 10 min and 40 min after intravenous injection. Plasma ketamine concentrations were measured by a gas chromatography mass spectrometer coupled to mass detector (Hewlett Packard GC 6890 with MS 5973) equipped with an autosampler and a HP-5MS capillary column (12.5 m × 0.20 mm i.d. 0.33 μm film thickness) (Agilent Technologies, Palo Alto, CA) was used for GC-MS analysis [12]. Blood ethanol concentrations were determined by an alcohol diagnostic kit available commercially (Diagnostic Chemicals Limited, Oxford, CT); the rate of increase in absorbance at 340 nm is recorded with a spectrophotometer (Beckman DU650).

### Intrathecal administration and blood pressure measurement

Procedures for intrathecal administration to anesthetized rats were similar to those described previously [10,13]. The rats were anaesthetized with urethane (1.2 g/kg, i.p.). Additional urethane (0.3 g/kg, i.p.) was applied if the rats responded to tail pinch or to intrathecal insertion of polyethylene tubing. The left femoral artery was cannulated with a polyethylene tubing (PE 50) and connected to a pressure transducer with its output to a Gould EasyGraf Recorder (TA420) for recording of blood pressure. The signals from the recorder were sent to a data acquisition system (MP 100, BIOPAC System, Inc.) for continuous recording of blood pressure, and the built-in function of the acquisition system provides simultaneous measurements of mean arterial pressure (MAP). The right femoral vein was cannulated for intravenous injection of ketamine or ethanol. Ketamine (1 ml/kg) at known concentrations was injected into the femoral vein in 100 seconds. Rats were mounted in a stereotaxic header and implanted with a spinal catheter for intrathecal injection. A slit was made in the atlanto-occipital membrane and the catheter (PE-10 tubing) was inserted down into the spinal subarachnoid space so that the tip was placed in the vicinity of T7-T9 segment. The reagents at known concentrations were injected intrathecally at a volume of 10 μL, which was followed by 10 μL of saline to wash in the agent. As a negative control, intrathecal saline did not elicit any significant changes in blood pressure. NMDA was applied at intervals of 30 min. NMDA or other chemicals at known concentrations were dissolved in saline and injected with a microsyringe pump (KDS 100). After NMDA-induced responses were stable over two consecutive tests, experiments were then carried out. Firstly, to examine whether ketamine or ethanol affected NMDA-induced responses, ketamine or ethanol was injected intravenously 10 min before the next application of NMDA. Secondly, to examine the effects of ethanol on ketamine inhibition of NMDA-induced responses, ketamine was applied immediately or at 10 min or 30 min after intravenous injection of ethanol; NMDA was applied 10 min after administration of ketamine. Thirdly, to examine the effects of pretreatment with PKA activators on modulation of ketamine effects, the activators was applied intrathecally 10 min prior to intravenous ketamine; NMDA was applied 10 min and 40 min after administration of ketamine.

### Western blot analysis

The procedure for Western blot analysis of spinal cord tissue was similar to that described in earlier studies [11,14]. Rats were anaesthetized by intraperitoneal injection of urethane (1.2 g/kg). A group of 3 rats was used to determine the levels of several phosphorylated residues on NMDA receptor subunits following administration of ethanol or PKA activators. For determination of the effects of ethanol, a control rat was sacrificed after the surgery without administration of ethanol; one rat each was sacrificed at 10 and 30 min following intravenous injection of ethanol. For each dose tested, the above experiments were repeated 4-5 times. For determination of the effects of PKA activators, a control rat was sacrificed at 10 min after intrathecal injection of saline; one each rat was sacrificed at 10 min after intrathecal injection of cAMPS-Sp (0.5 and 5 nmol). The thoracic segments of spinal cord were removed immediately after cardiac perfusion with normal saline containing protease inhibitors (Complete protease inhibitor cocktail tablets, Roche Diagnostics, GmbH). Coronal 1000 μm-thick sections from T7-T9 segments of spinal cord were prepared and quickly frozen by cold spray (FREEZE 75; CRC Industry Europe NV, Zele, Belgium). The lateral horn regions of the slices from each rat were punched out by a tissue puncher (0.75 mm in diameter). The isolated tissues were frozen in liquid nitrogen and stored at -85°C until use. The tissue was homogenized in 60 μL of lysis buffer (0.32 M sucrose, 1 mM EDTA and 1 mTIU·mL-1 aprotinin) with a homogenizer (Glas-Col, Terre Haute, IN) under ice bath. SDS was added to the sample to a final concentration of 0.1%, and 20 μg of protein was electrophoresed and transferred onto a nitrocellulose (NC) membrane. After NC membrane blocking and washing, blots were probed with primary antibody, rabbit anti-GluN1 polyclonal antibody (1:800, Upstate Biotechnology Inc., Lake Placid, NY, USA) and rabbit anti-pGluN1 antisera (serine 896, 1:1500 and serine 897, 1:1500, Upstate Biotechnology Inc.) in TBS-T containing 5% skimmed milk powder overnight at 4°C on a 2D shaker. The blot was then incubated with secondary goat anti-rabbit (1:2000, Santa Cruz Biotechnology Inc., Santa Cruz, CA, USA) antibody conjugated to horseradish peroxidase, which was measured with Western Blotting Luminol Reagent (Santa Cruz Biotechnology, Santa Cruz, CA). The specific protein bands were visualized using the enhanced chemiluminescence (ECL) reagents. The chemiluminescent signal was detected by X-ray film (Fuji Photo Film Co., Ltd., Tokyo), and the intensity of the bands was digitalized by scanner and analysed with UN-SCAN-IT gel software version 6.1 for Windows (Silk Scientific Corporation, Orem, UT, USA). Protein concentrations were determined by bicinchoninic acid method (Sigma Co.) using bovine albumin as standard.

### Chemicals and statistical analysis

cAMPS-Sp triethylammonium salt, a PKA activator, was obtained from Tocris Cookson Ltd. (Bristol, UK). Ethanol was purchased from Riedel-de Haen (Deisenhofen, Germany). NMDA, ketamine, aprotinin and other reagents used for Western blot analysis were purchased from Sigma Co. (St. Louis, Missouri, USA). Stock solutions were prepared in distilled water; further dilutions were made in saline. The reagents for electrophoresis were obtained from Bio-Rad Laboratories (Richmond, CA).

Data are presented as mean ± SEM and were plotted and analysed statistically with GraphPad Prism version 4.0 for Windows, GraphPad Software (San Diego, CA). The time-effect relationship of ethanol or ketamine on NMDA-induced pressor responses was analysed using repeated measure ANOVA followed by Newman-Keuls post-test. The effects of prior administration of ethanol or pretreatment with cAMPS-Sp on ketamine action at different times after administration of ketamine were analysed using two-way ANOVA followed by Bonferroni post-test. The statistical evaluation of western blots was analysed using one-way ANOVA followed by Newman-Keuls post-test. P < 0.05 was considered statistically significant.

## Results

### Ketamine inhibition of spinal NMDA-induced pressor responses

**Figure 1:**
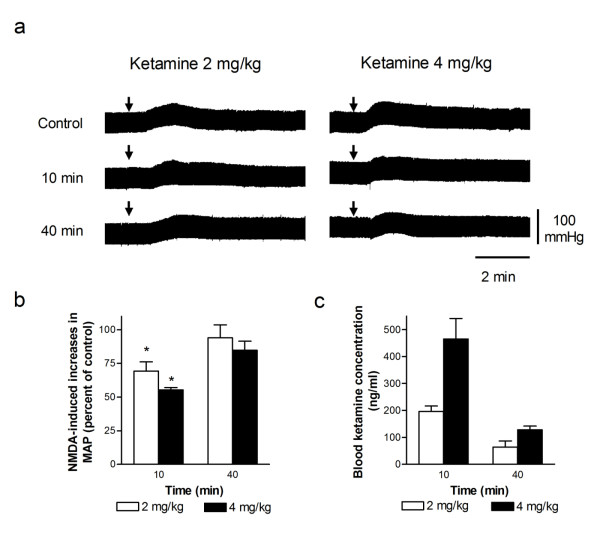
**Intravenous injection of ketamine dose-dependently inhibited pressor effects induced by intrathecal NMDA**. (a) Representative recordings of changes in blood pressure induced by an intrathecal injection of NMDA (2 nmol, indicated by an arrow) and the changes in NMDA-induced pressor responses at 10 and 40 min after a single intravenous injection of ketamine (2 or 4 mg/kg). NMDA was applied intrathecally every 30 min. (b and c) Bar graphs show the time course of the percentage changes in NMDA-induced increases in mean arterial pressure (MAP, b) and blood ketamine concentration (c) following intravenous injection of two doses of ketamine (2 and 4 mg/kg). The peak magnitude of NMDA-induced increase in MAP immediately prior to application of ketamine is taken as control (100%).*Significant difference from control analyzed using the repeated measure ANOVA followed by Newman-Keuls post-test.

Resting mean arterial pressure (MAP) of the urethane-anesthetized rats was 80.2 ± 4.3 mmHg (n = 26). As in our previous study [10], the MAPs increased following intrathecal injection (1, and 2 nmol, 10 μL) of NMDA in a dose-dependent manner, which were 14.3 ± 2.5 (n = 6), and 26.5 ± 2.3 (n = 16) mmHg in magnitudes following the applications of 1 and 2 nmol of NMDA, respectively. Consecutive intrathecal administration of NMDA (2 nmol) at intervals of 30 min elicited reproducible increase in MAP. Intravenous injection of ketamine (2 and 4 mg/kg) alone did not cause significant changes in MAP. However, NMDA-induced pressor effects were dose-dependently attenuated by intravenous administration of ketamine. Representative recording of ketamine inhibition of spinal NMDA-induced pressor response is illustrated in Figure 1a. The time course of percentage changes in NMDA-induced pressor responses and the corresponding blood concentrations of ketamine are illustrated in Figure 1b and 1c, respectively. The decline level of blood ketamine over time was accompanied by a comparable degree of reductions in ketamine inhibition of pressor responses induced by NMDA after a single injection of ketamine; NMDA-induced pressor responses decreased by 31% and 45% when blood ketamine concentrations were about 195 and 460 ng/mL at 10 min after intravenous injection of 2 mg/kg and 4 mg/kg of ketamine, respectively.

### Ethanol inhibition of spinal NMDA-induced pressor responses

**Figure 2:**
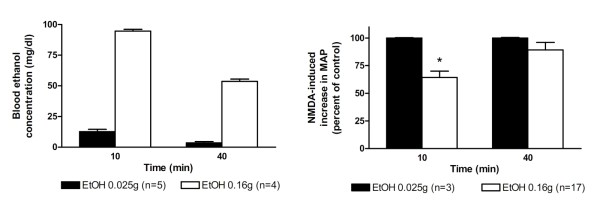
**Bar graphs show the time course of blood ethanol concentrations (left part) and percentage changes in NMDA-induced increases in MAP (right part) at 10 min and 40 min following intravenous injection of two doses of ethanol (0.025 g and 0.16 g)**. The peak magnitude of NMDA-induced increase in MAP immediately prior to application of ethanol is taken as control (100%). *Significant difference from control analyzed using the repeated measure ANOVA followed by Newman-Keuls post-test.

Similar to the results in our previous reports [10,11], spinal NMDA-induced pressor responses were dose-dependently attenuated by intravenous administration of a bolus of ethanol (Figure 2). Intravenous injection of low dose of ethanol (0.025 g) did not caused significant changes in NMDA-induced pressor effects at 10 min after injection when blood ethanol concentration was about 12.7 mg/dL. NMDA-induced pressor responses decreased by 36% when blood ethanol concentrations were about 94.6 mg/dL at 10 min after intravenous injection of higher dose of ethanol (0.16 g). The decline level of blood ethanol over time was accompanied by a comparable degree of reductions in ethanol inhibition of NMDA-induced pressor responses.

### Ethanol potentiated ketamine inhibition of spinal NMDA-induced pressor responses

**Figure 3:**
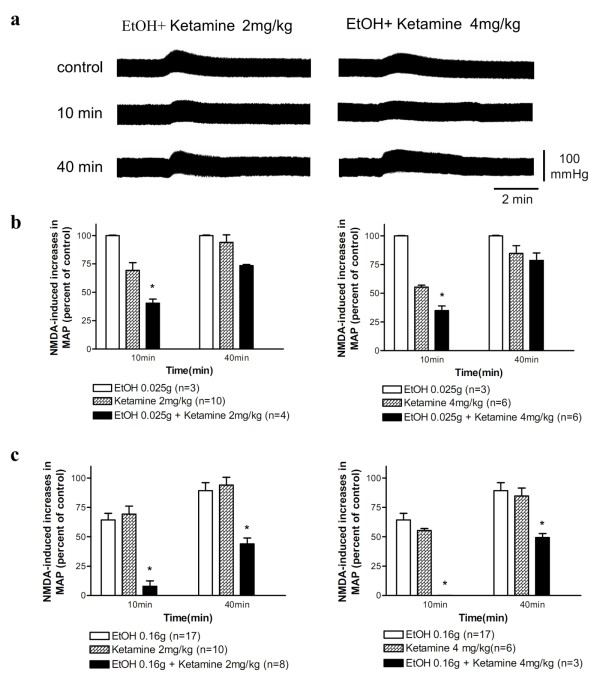
**(a) Representative recordings of changes in blood pressure induced by an intrathecal injection of NMDA (2 nmol) and the changes in NMDA-induced pressor effects at 10 and 40 min after an intravenous injection of ethanol (EtOH, 0.025 g) immediately followed by ketamine (2 mg/kg, left panel; 4 mg/kg, right panel)**. (b) Bar graphs show percentage changes in NMDA-induced increases in MAP at 10 min and 40 min after intravenous injection of ethanol (0.025 g) alone, ketamine alone (2 mg/kg, left; 4 mg/kg, right), and ethanol (0.025 g) immediately followed by ketamine (2 mg/kg, left; 4 mg/kg, right). The peak magnitude of NMDA-induced increase in MAP immediately prior to application of ethanol and/or ketamine is taken as control (100%). (c) Similar to (b) except that higher dose (0.16 g) of ethanol was injected. *P < 0.05 versus ethanol or ketamine alone group analyzed using two-way ANOVA followed by Bonferroni post-test.

Ethanol (0.025 g) applied alone had little effects on NMDA-induced responses. However, ethanol (0.025 g) significantly potentiated ketamine inhibition of NMDA-induced pressor effects when ethanol was applied just before ketamine (Figure 3a, b). Intravenous injection of ethanol (0.16 g) followed by ketamine (2 or 4 mg/kg) caused a strong inhibition of NMDA-induced pressor effects at 10 min after the injection (Figure 3c); the degree of inhibition induced by combined administration of ethanol and ketamine was greater than the sum of that induced by ethanol or ketamine alone (Figure 3b, c). These results revealed that ethanol synergistically potentiated ketamine inhibition of NMDA-induced responses.

### Ketamine inhibition of spinal NMDA-induced pressor responses was reduced at 10 min after administration of ethanol

**Figure 4:**
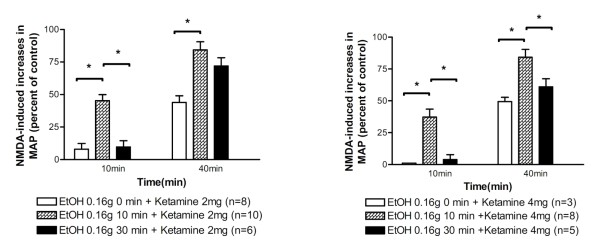
**Bar graphs show percentage changes in NMDA-induced increases in MAP 10 min and 40 min after intravenous injection of ketamine (2 mg/kg, left part; 4 mg/kg, right part) applied immediately (0 min) or at 10 min and 30 min after intravenous injection of ethanol (0.16 g)**. The peak magnitude of NMDA-induced increase in MAP immediately prior to application of ethanol and/or ketamine is taken as control (100%). *Statistically significant difference between groups analyzed using two-way ANOVA followed by Bonferroni post-test.

The degree of inhibition by ketamine (2 and 4 mg/kg) of NMDA-induced pressor effects was examined immediately (0 min) or at 10 min or 30 min after intravenous injection of ethanol (0.16 g). The degree of ketamine inhibition of NMDA-induced pressor effects at 10 min after intravenous ethanol was significantly less than that at 0 and 30 min (Figure 4). NMDA-induced responses decreased by 92%, 55%, and 90% while ketamine (2 mg/kg) was applied at 0 min, 10 min, and 30 min after injection of ethanol (0.16 g), respectively; NMDA-induced responses decreased by 100%, 63%, and 96% while ketamine (4 mg/kg) was applied at 0 min, 10 min, and 30 min after ethanol (0.16 g), respectively. These results showed a reduction of ketamine and/or ethanol inhibition at 10 min after administration of ethanol.

### Ethanol increased the levels of phosphoserine 897 on the GluN1 subunit in the lateral horn regions of spinal cord at 10 min after administration

**Figure 5:**
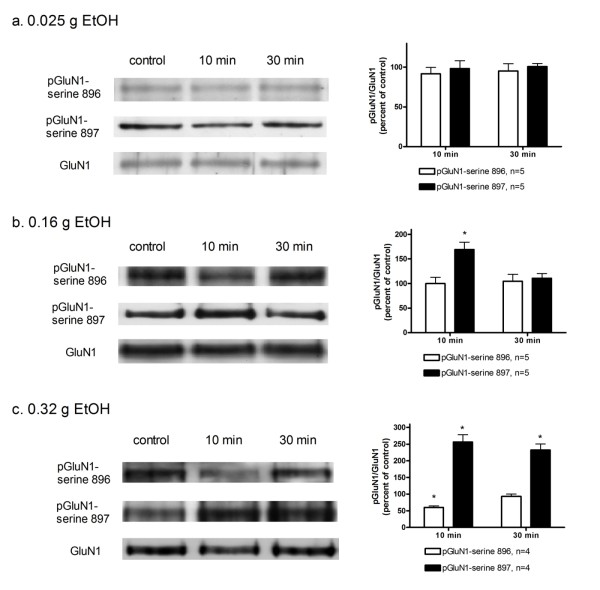
**(a) Left panel: western blot analysis of the levels of phosphoserine 896 on GluN1 subunit (pGluN1-serine 896), phosphoserine 897 on GluN1 subunit (pGluN1-serine 897), and GluN1 subunit (GluN1) in lateral horn regions of T7-T9 segments of rat spinal cord without (control) and at 10 min and 30 min after intravenous injection of ethanol (EtOH, 0.025 g)**. The percentage changes in the ratio of pGluN1 (serine 896 and serine 897) to GluN1 are shown in the right panel. The ratio of pGluN1 to GluN1 in rats without administration of ethanol is taken as control (100%). Panels (b) and (c) are similar to panel (a) except that 0.16 g and 0.32 g ethanol were injected, respectively. Values denote mean + SEM from 4-5 separate experiments. *Statistically significant difference from control analyzed by one-way ANOVA followed by Newman- Keuls post-test.

We used western blot analysis to determine the levels of phosphoserine 896 on GluN1 subunit (pGluN1-serine 896) and phosphoserine 897 on GluN1 subunits (pGluN1-serine 897), and the protein contents of GluN1 subunits in the lateral horn regions of spinal cord. Intravenous injection of ethanol (0.025 g) had little effects on the levels of pGluN1-serine 897 (Figure 5a). Higher doses of ethanol (0.16 g and 0.32 g) caused a significant increase in the level of pGluN1-serine 897 in a dose-dependent manner at 10 min after the administration of ethanol (Figure 5b, c); the increase lasted for over 30 min following administration of high dose (0.32 g) of ethanol. The level of pGluN1-serine 896 did not change over time after administration of lower doses of ethanol (0.025 g, 0.16 g), but decreased at 10 min after administration of high dose (0.32 g) of ethanol (Figure 5).

### Intrathecal a PKA activator, at doses increased the levels of pGluN1-serine 897, blocked ketamine inhibition

**Figure 6:**
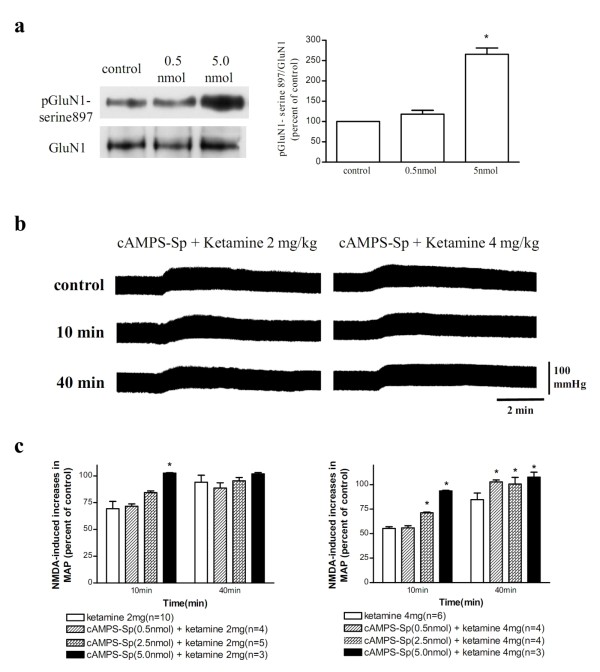
**(a) Left panel: Western blot analysis of the levels of phosphoserine 897 on the GluN1 subunit (pGluN1-serine 897, top) and GluN1 subunit (GluN1, bottom) in lateral horn regions of spinal cord at 10 min after intrathecal injection of saline (control) and cAMPS-Sp (0.5 and 5 nmol)**. The percentage changes in the ratio of pGluN1-serine 897 to GluN1 are shown in the right panel. Values denote mean + SEM from three separate experiments. *Significant difference from control (100%). (b) Representative recordings of changes in blood pressure induced by an intrathecal injection of NMDA (2 nmol) before (control) and at 10 min and 40 min after intravenous injection of ketamine (2 mg/kg, left panel; 4 mg/kg, right panel) following pretreatment with cAMPS-Sp (5 nmol); cAMPS-Sp was applied intrathecally 10 min before the administration of ketamine. (c) Bar graphs show percentage changes in NMDA-induced increases in MAP 10 min and 40 min after intravenous injection of ketamine (2 mg/kg, left part; 4 mg/kg, right part) following intrathecal pretreatment with various doses of cAMPS-Sp. *Statistically significant difference from ketamine alone group (without pretreatment with cAMPS-Sp) analyzed using two-way ANOVA followed by Bonferroni post-test.

Intrathecal cAMPS-Sp (0.5 nmol), a PKA activator, had no significant effects on the levels of pGluN1-serine 897 in the lateral horn of the spinal cord at 10 min post-injection; Higher dose of cAMPS-Sp (5 nmol) significantly increased the levels of pGluN1-serine 897 (Figure 6a). Intrathecal injection of cAMPS-Sp (5 nmol) had negligible effects on blood pressure and on spinal NMDA-induced pressor responses applied 10 min before administration of NMDA (n = 3). However, intrathecal injection of cAMPS-Sp 10 min before intravenous injection of ketamine dose-dependently blocked the inhibitory effects of ketamine on spinal NMDA-induced pressor responses (Figure 6b, c).

## Discussion

The present study showed for the first time that ethanol differentially regulate, depending on ethanol exposure time and the underlying changes in the levels of pGluN1-serine 897, ketamine inhibition of spinal NMDA receptor-mediated responses. In addition, our study provided the first evidence that an increase in the levels of pGluN1-serine 897 reduced the inhibitory potency of ketamine on NMDA receptor function.

Our previous study found that intravenous ethanol, at doses without noticeable effects on intrathecal AMPA-mediated pressor responses, significantly inhibited NMDA-mediated pressor responses, indicating NMDA receptors are main targets of ethanol in sympathetic neurons of spinal cord [10]. The present study demonstrated that intravenous ethanol inhibited spinal NMDA-induced pressor responses as in our previous findings. In addition, we also found that intravenous ketamine inhibited NMDA-induced pressor effects in a blood concentration-dependent and reversible manner. An interesting finding was that intravenous injection of ethanol just before ketamine may produce synergetic effects on the inhibition of NMDA-induced responses, i.e. the combined inhibition is greater than the sum of individual inhibition. The synergistic inhibition may be of greater concern with respect to fetal brain damage because NMDA receptors play an important role during the developmental period of synaptogenesis [15,16]. Because of the ability of ketamine to produce mild-altering effects it is frequently abused. Abuse of ketamine mixed with other drugs is increasingly common in rave party [17,18], where intake of ketamine would be combined with that of ethanol. There were reports of the lethality from mixed-drug intoxication involving ketamine and ethanol [19]. The current findings showing a potentiated inhibition of ketamine by ethanol on NMDA receptor function may provide a molecular mechanism for the neurotoxicity induced by combined intake of ethanol and ketamine.

By utilizing a patch clamp technique to examine NMDA-activated currents, ketamine may inhibit the NMDA receptor by two distinct mechanisms. First, ketamine blocks the open channel and thereby reduces channel mean open time; secondly, ketamine may bind to the closed receptor and decrease the frequency of channels opening by an allosteric mechanism. Low concentrations of ketamine predominantly caused closed-channel blockade, whereas both open and closed channel blockade occurred at higher ketamine concentration [20]. Ethanol is well known to inhibit NMDA receptor function. However, the precise site and the action mechanism underlying alcohol inhibition have not been established [21]. Ethanol inhibits NMDA receptors in a noncompetitive manner and does not appear to act by interfering with either glutamate or glycine binding. By studying cells transfected with NMDA receptor subunits, acute alcohol treatment has been shown to interact with several transmembrane domains of NMDA receptors because alterations in certain amino acids of NMDA receptor subunits changed alcohol sensitivity [22,23]. Alcohol may also act at a site located in a domain exposed to the extracellular environment [24]. In addition, ethanol sensitivity of NMDA receptors may be modulated by discrete sites within the TM3 and TM4 domains of the GluN1 subunit [25]. These studies suggest a putative binding site of NMDA receptor to ethanol. The current study demonstrated that ethanol may synergistically potentiate ketamine inhibition of NMDA-induced responses, suggesting that the binding site of ethanol may be different to that of ketamine in NMDA receptors and both binding sites may interact with each other in regulating NMDA receptor function. Actually, a recent study also showed that ifenprodil, a selective NMDA receptor GluN2B subunit antagonist, may enhance the anti-hyperalgesic effect of ketamine [26].

Phosphorylation is important in direct and indirect modulation of NMDA receptors. The serine residue 897 on GluN1 subunit is specifically phosphorylated by PKA; the serine residue 890 and 896 is phosphorylated by PKC; the tyrosine residue 1336 on GluN2B subunit (pGluN2B-tyrosine 1336) is specifically phosphorylated by protein tyrosine kinases [27]. Acute and chronic exposure of ethanol affects the function of specific intracellular signaling pathways, including PKA, PKC, and tyrosine kinase signaling pathways [28,29]. Some kinases such as fyn tyrosine kinase, PKC and PKA are able to reduce NMDA receptor sensitivity to acute ethanol [30-32]. Our previous study demonstrated that continuous intravenous ethanol infusion may activate PKA, PKC, and Src tyrosine kinase leading to increases in the levels of pGluN1-serine 896, pGluN1-serine 897, and pGluN2B-tyrosine 1336, respectively, in the lateral horn of the spinal cord [11]. We also showed that the increase in the levels of pGluN1-serine 896 and pGluN2B-tyrosine 1336, but not pGluN1-serine 897, may counteract ethanol inhibition of spinal NMDA-induced pressor responses and be responsible for acute ethanol tolerance during prolonged ethanol exposure. In the current study, we found that a single bolus intravenous injection of low dose of ethanol would cause an increase in the levels of pGluN1-serine 897, but not pGluN1-serine 896, a short period of time (10 min) after the injection when blood ethanol concentrations were about 90 mg/dL (0.09%). The increases in the levels of pGluN1-serine 897 induced by ethanol may reduce the inhibitory potency of ketamine as supported by our results showing that intrathecal cAMPS-Sp, a PKA activator, at doses elevating the levels of pGluN1-serine 897 significantly blocked ketamine inhibition of spinal NMDA-induced responses. The results provide the first *in vivo *evidence that PKA signaling pathways may participate in the regulation of ketamine inhibition of NMDA receptor function. In addition, our results have an important implication that alteration of the phosphorylated levels of NMDA receptor subunits may have influence on the effects of NMDA receptor antagonists. A recent study showed that inhibition of cAMP hydrolysis by phosphodiesterase inhibitors significantly reversed ketamine-induced anesthesia in mice [33], further supporting the idea that cAMP signaling pathways are involved in the regulation of ketamine effects.

Our previous study showed that the immunoreactivity to pGluN1-serine 896 (regulated by PKC) was decreased at 10 min but increased at 40 min in neurons of intermediolateral cell column following continuous ethanol infusion [11]. The present study also showed that pGluN1-serine 896 was significantly reduced in the lateral horn regions of spinal cord at 10 min after administration of higher dose of ethanol. Ethanol is widely reported to regulate the function of PKC [34,35]. PKC may regulate NMDA receptor function through non-receptor tyrosine kinase [36]. It has been suggested that ethanol-induced tyrosine dephosphorylation of NMDA receptor subunits plays an important role in mediating the inhibitory effects of ethanol on NMDA receptor function [37]. It is possible that decreases in the levels of pGluN1-serine 896 may contribute to the inhibitory effects of ethanol on spinal NMDA-induced responses. Further work is required to establish this. In addition to NMDA receptors, GABA receptors are another important target for ethanol action. Ethanol increases GABA receptor function in several brain regions [3,38]. Ethanol inhibition of NMDA receptor activity has been suggested to be both directly through actions on the NMDA receptors, and indirectly, possibly through potentiation of GABA receptor activity [39]. Therefore, the possibility that ethanol regulation of NMDA-induced responses was secondary to the effects of ethanol on other targets such as GABA receptors cannot be ruled out.

Heavier alcohol consumption is associated with cardiovascular dysfunction such as hypertension and stroke [40]. However, epidemiological studies have also showed that moderate consumption of alcohol is associated with reduced risk of coronary artery disease [41]. A positive relationship between alcohol consumption and blood pressure is well-established in epidemiologic studies [42,43]. Though the mechanisms underlying alcohol-induced hypertension remain unclear, alteration of central sympathetic activity has been suggested to participate in alcohol-induced changes in blood pressure [44,45]. It is likely that differential regulation by ethanol of NMDA receptor function in spinal sympathetic neurons may contribute to ethanol regulation of cardiovascular function, although further studies are required to clarify this.

## Conclusion

In summary, our results indicate that simultaneous intake of ethanol and ketamine may produce synergistically inhibitory effects on NMDA receptor function. However, intake of ethanol a certain period of time prior to ketamine may reduce the inhibitory effects of ketamine by activating PKA signaling pathways resulting in elevated levels of pGluN1-serine 897.

## Competing interests

The authors declare that they have no competing interests.

## Authors' contributions

The experiments of the study were carried out largely by NT, under the instructions of HH and CC. NT drafted the manuscript. HR participated in the determination of ketamine levels. WK participated in Western blot analysis. HH participated in the design of the study and provided professional suggestions regarding the experiments and manuscripts. CC conceived of the study, and participated in its design and coordination. All authors read and approved the final manuscript.
